# Ecdysone Receptor (EcR) Is Involved in the Transcription of Cell Cycle Genes in the Silkworm

**DOI:** 10.3390/ijms16023335

**Published:** 2015-02-03

**Authors:** Wenliang Qian, Lixia Kang, Tianlei Zhang, Meng Meng, Yonghu Wang, Zhiqing Li, Qingyou Xia, Daojun Cheng

**Affiliations:** State Key Laboratory of Silkworm Genome Biology, Southwest University, Chongqing 400715, China; E-Mails: wenliang20081103@126.com (W.Q.); hnklx1986@163.com (L.K.); zhangtianlei2011@sina.com (T.Z.); keven190@sina.com (M.M.); wangyonghu2006@163.com (Y.W.); lizhiqing@swu.edu.cn (Z.L.)

**Keywords:** EcR, ecdysone, *E2F-1*, *CycE*, transcription

## Abstract

EcR (ecdysone receptor)-mediated ecdysone signaling pathway contributes to regulate the transcription of genes involved in various processes during insect development. In this work, we detected the expression of *EcR* gene in silkworm ovary-derived BmN4 cells and found that *EcR* RNAi result in an alteration of cell shape, indicating that EcR may orchestrate cell cycle progression. *EcR* RNAi and *EcR* overexpression analysis revealed that in the cultured BmN4 cells, EcR respectively promoted and suppressed the transcription of *E2F-1* and *CycE*, two genes controlling cell cycle progression. Further examination demonstrated that ecdysone application in BmN4 cells not only changed the transcription of these two cell cycle genes like that under *EcR* overexpression, but also induced cell cycle arrest at G2/M phase. *In vivo* analysis confirmed that *E2F-1* expression was elevated in silk gland of silkworm larvae after ecdysone application, which is same as its response to ecdysone in BmN4 cells. However, ecdysone also promotes *CycE* transcription in silk gland, and this is converse with the observation in BmN4 cells. These results provide new insights into understanding the roles of EcR-mediated ecdysone signaling in the regulation of cell cycle.

## 1. Introduction

Ecdysone receptor (EcR), as a member of nuclear receptor family, was identified and well-characterized in insects and primarily contributes to meditate the signaling of steroid hormone ecdysone [1,2,3]. Generally, ecdysone directly binds to the heterodimer comprising of EcR and Ultraspiracle (USP) and subsequently activates a transcriptional response of its primary and secondary response genes [4,5]. More evidence supports that EcR-mediated ecdysone pathway is required for all aspects of insect growth and development, including developmental timing [6], embryogenesis [7], larval molting [8], degeneration, remodeling or specification of organs during metamorphosis [9], and reproduction [10].

Cell cycle-modulated cell growth and cell proliferation have been shown to connect to the growth and development of multicellular organisms including insects [11,12,13,14]. Extensive studies provided a comprehensive understanding that cell cycle is principally and directly governed by the paired members from several protein families of cyclins, cyclin-dependent kinases (CDK), and cdk inhibitors [15,16]. It is conservation among multicellular organisms that cyclin E (CycE) and its CDK partner Cdk2 are responsible for initiating the G1/S transition by promoting DNA replication [17], and CycB interacts with Cdk1 to promote the G2/M transition by triggering mitosis [18,19]. In addition, both *CycE* and *CycB* are transcriptionally regulated by E2F-1 protein [20,21].

Recently increasing reports have shown that ecdysone signal also plays roles in the regulation of cell cycle progression in insects [22,23,24,25,26]. In the fruit fly (*Drosophila melanogaster*), ecdysone and its receptor EcR modulate cell cycle progression in wing disc by repressing the expression of *Wingless* (*Wg*) gene [27,28]. Wingless acts to down-regulate the key cell cycle genes, such as *E2F-1* and *cycE* [29,30]. However, to date, the mechanism of ecdysone regulation of cell cycle progression in insects is poorly understood.

From our previously obtained microarray data of gene expression in cultured silkworm (*Bombyx mori*) ovary-derived BmN4-SID1 cells [31], we found that *EcR* gene revealed a weak expression in BmN4-SID1 cells, indicating that EcR may be also involved in the regulation of the transcription of cell cycle genes in silkworm cells. Here, we performed RNA interference (RNAi)-mediated knockdown of *EcR* gene and ecdysone treatment in the silkworm at cellular and individual scales, and found that EcR-mediated ecdysone signaling can regulate the transcription of two cell cycle genes, *E2F-1* and *CycE*.

## 2. Results

### 2.1. EcR Was Expressed in Silkworm BmN4 Cells

**Figure 1:**
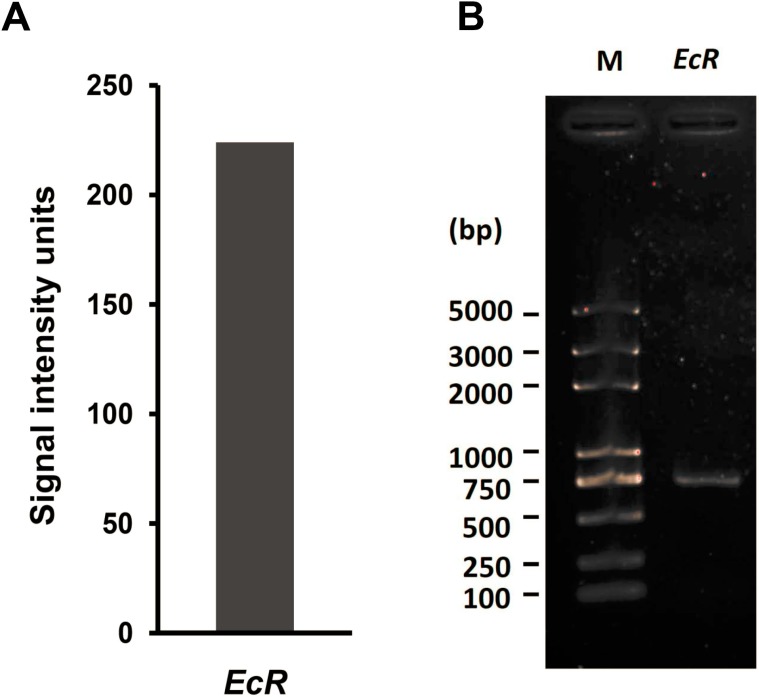
*EcR* expression in BmN4 cells. (**A**) Microarray data of mRNA expression of silkworm *EcR* gene in BmN4 cells; (**B**) Quantitative RT-PCR detection of mRNA expression of silkworm *EcR* gene in BmN4 cells. M: Molecular weight marker.

We previously profiled the genome-wide expression in cultured silkworm ovary-derived BmN4 cells by using microarray approach [31]. From the related microarray data (GSE no.: GSE34246), we observed that the expression signal intensity of two probes (sw11842 and sw11434) for silkworm *EcR* gene exceeded a value of 200 units (Figure 1A), suggesting that *EcR* gene is likely expressed in cultured BmN4 cells. Quantitative RT-PCR examination confirmed an obvious expression of *EcR* gene in BmN4 cells (Figure 1B). Together with the observation that *EcR* expression could be detected in cultured mosquito (*Aedes albopictus*) cells [32], we speculated that EcR may be involved in cell cycle or cell growth in cultured cells without the existence of ecdysone.

### 2.2. EcR RNAi Alters the Shape of Silkworm BmN4-SID1 Cells

**Figure 2:**
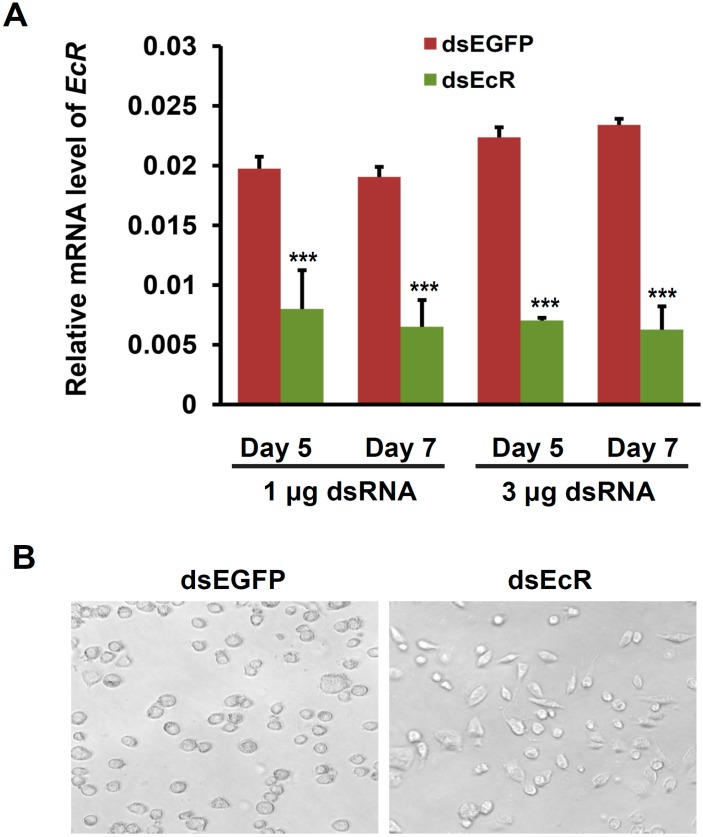
*EcR* RNAi changes the shape of BmN4-SID1 cells. (**A**) Quantitative RT-PCR assay of RNAi-based knockdown efficiency of *EcR* expression in silkworm BmN4-SID1 cells. *EGFP* RNAi was used as control. Error bars represents mean and S.D., *** *p* < 0.001, compared with the control; (**B**) Effects of *EcR* RNAi on the shape of BmN4-SID1 cells. The BmN4-SID1 cells were checked on the seventh day after the treatment with different dsRNA (dsEcR for *EcR* gene or dsEGFP for *EGFP* gene) using microscope.

In order to ascertain the roles of EcR in BmN4 cells, we performed a RNAi experiment of *EcR* gene in cultured BmN4-SID1 cells, which is established by overexpressing the *SID1* gene, a gene with high efficiency in the uptake of exogenous double strand RNA (dsRNA) into host cells, in BmN4 cells [33]. The dsRNAs targeting the *EcR* gene and *EGFP* (enhanced green fluorescent protein) gene as control were separately transfected into BmN4-SID1 cells in a dosage of either 1 or 3 μg per plate well. Quantitative RT-PCR analysis showed that compared with the control of *EGFP* dsRNA treatment, *EcR* expression was remarkably silenced at both the fifth and seventh day after the treatment with *EcR* dsRNAs (Figure 2A). Further microscopy analysis found that the morphology of the BmN4-SID1 cells was transformed into fusiform from roundness (Figure 2B). This observation is similar to the morphological response of the fruit fly Kc Cells to ecdysone [25], indicating that cell cycle progression of the BmN4-SID1 cells was changed after *EcR* RNAi.

### 2.3. RNAi or Overexpression of EcR Gene Disrupts the Expression of Cell Cycle Genes in BmN4 Cells

**Figure 3:**
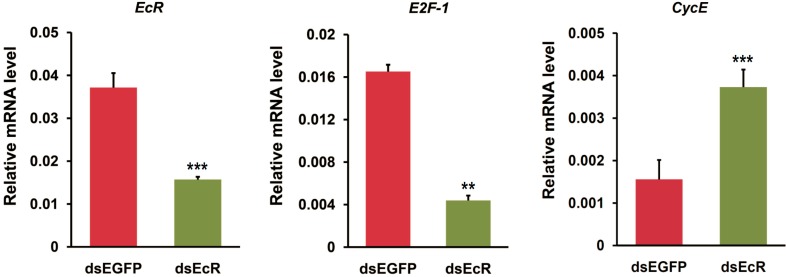
*EcR* RNAi changes the expression of cell cycle genes in BmN4-SID1 cells. The expression changes of cell cycle genes (*E2F-1* and *CycE*) after the RNAi of *EcR* and *EGFP* in silkworm BmN4-SID1 cells were examined using quantitative RT-PCR. Error bars represents mean and S.D., ** *p* < 0.01, *** *p* < 0.001, compared with the control.

Given that *EcR* RNAi changed the shape of BmN4 cells and may alter cell cycle progression, we proposed that EcR may be involved in regulating the expression of cell cycle genes. Here, we focused on two DNA replication-related genes, *E2F-1* and *CycE*, and checked whether their expressions could be changed after *EcR* RNAi. As expected, quantitative RT-PCR examination showed that in BmN4-SID1 cells after *EcR* RNAi, *E2F-1* and *CycE* were down- and up-regulated, respectively (Figure 3). This is obviously different with the previous observation in the fruit fly wing that *CycE* expression is positively regulated by E2F-1 [20,21].

**Figure 4:**
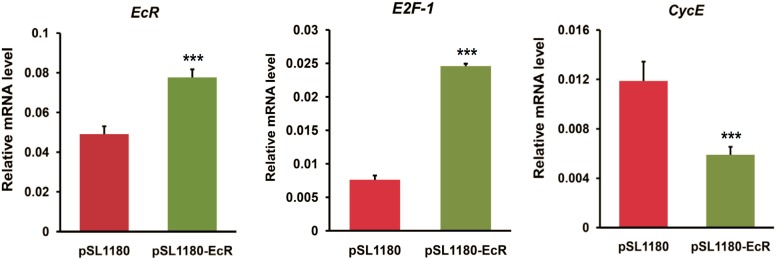
*EcR* overexpression disrupts the expression of cell cycle genes in BmN4 cells. The expression changes of cell cycle genes (*E2F-1* and *CycE*) after the overexpression of *EcR* gene in silkworm BmN4 cells were examined using quantitative RT-PCR. Expression vector excluding *EcR* gene was used as control. Error bars represents mean and S.D., *** *p* < 0.001, compared with the control.

To further confirm the roles of EcR in the transcription of *E2F-1* and *CycE*, we performed an overexpression of *EcR* gene in BmN4 cells. As shown in Figure 4, *E2F-1* expression was promoted following *EcR* overexpression. Conversely, *CycE* expression was inhibited after *EcR* overexpression. This is contrary to the effects of *EcR* RNAi on the transcription of both *E2F-1* and *CycE*.

### 2.4. Ecdysone Treatment Changes the Expression of Cell Cycle Genes in BmN4 Cells

**Figure 5:**
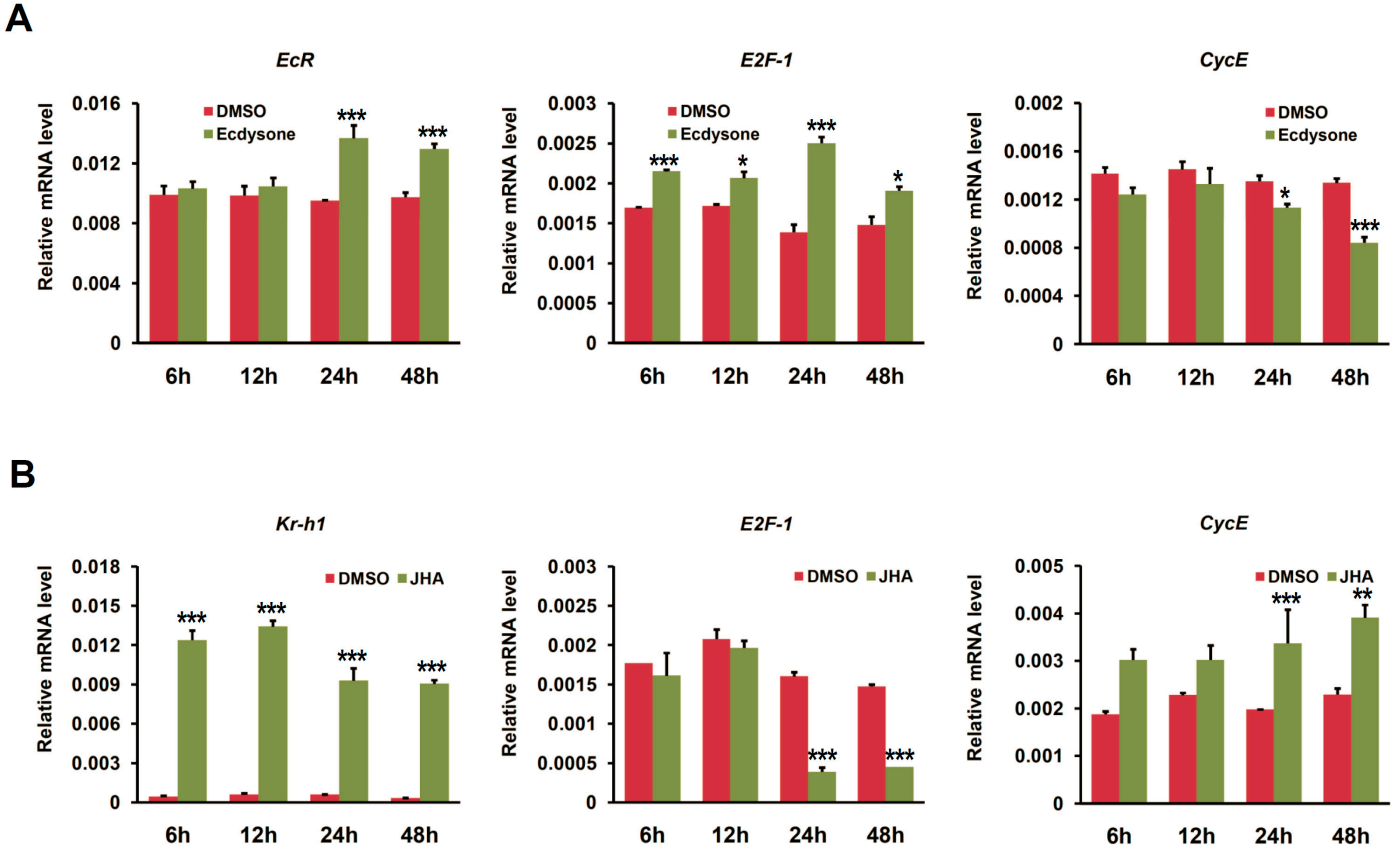
Time-dependent expression changes of cell cycle genes induced by ecdysone and JH Analog (JHA) in BmN4 cells. (**A**) The expression changes of cell cycle genes (*E2F-1* and *CycE*) at different time points, including 6, 12, 24, and 48 h, after ecdysone treatment in silkworm BmN4 cells were examined using quantitative RT-PCR. DMSO treatment was used as control. Error bars represents mean and S.D., * *p* < 0.05, ** *p* < 0.01, *** *p* < 0.001, compared with the control; (**B**) The expression changes of cell cycle genes (*E2F-1* and *CycE*) at different time points, including 6, 12, 24, and 48 h, after JHA treatment in silkworm BmN4 cells were examined.

Given that EcR is a key receptor in mediating ecdysone signaling in insects [1], we further examined the effects of ecdysone application on the expression of cell cycle genes in BmN4 cells. We firstly checked time-dependent effects of ecdysone treatment with a dosage of 1 μg per mL per plate well on the transcription of cell cycle genes. As shown in Figure 5A, *EcR* expression was significantly up-regulated after 24 h with ecdysone treatment. Further examination revealed that *E2F-1* expression was remarkably elevated at 6 h after ecdysone treatment and *CycE* was inhibited at 24 h (Figure 5A). In addition, given that the role of juvenile hormone (JH) is antagonistic to that of ecdysone in controlling developmental processes in insects [34,35], we also checked the effects of JH analog (JHA) on the expression of cell cycle genes in BmN4 cells. In our experiment, the dosage of JHA is 1 μg per plate well. Obviously, *Kr-h1* gene, a key transcription factor that is activated by JH and is involved in JH signaling [36,37], was up-regulated in BmN4 cells at 6 h after JHA treatment (Figure 5B). *E2F-1* and *CycE* were significantly down- and up-regulated after 24 h with JHA treatment, respectively, which is consistent with the observation in *EcR* RNAi, but converse with the effects of either *EcR* overexpression or ecdysone application.

**Figure 6:**
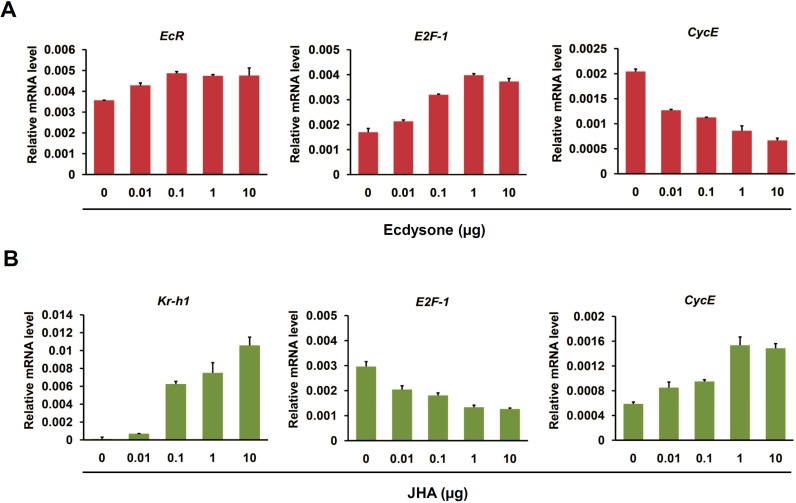
Dosage-dependent expression changes of cell cycle genes induced by ecdysone in BmN4 cells. (**A**) Dosage-dependent expression changes of cell cycle genes (*E2F-1* and *CycE*) after ecdysone treatment in silkworm BmN4 cells were examined using quantitative RT-PCR. Ecdysone treatment was initiated with different dosage, including 0, 0.01, 0.1, 1, and 10 µg per plate well. The cells were collected at 24 h after ecdysone treatment for further analysis; (**B**) Dosage-dependent expression changes of cell cycle genes (*E2F-1* and *CycE*) after the treatment of JHA as ecdysone antagonists were tested by quantitative RT-PCR. The analyzing approach for JHA treatment was same as ecdysone treatment.

We further investigated the effects of ecdysone and JHA treatment with different dosages on the transcription of cell cycle genes in BmN4 cells. As shown in Figure 6A, *EcR* expression was up-regulated at 24 h after ecdysone treatment with a dosage of 0.01 μg per plate well. *E2F-1* and *CycE* was elevated and inhibited after ecdysone treatment in a dosage-dependent manner, respectively (Figure 6A). Similarly, JHA treatment resulted in an up-regulation of *Kr-h1* and *CycE* but a down-regulation of *E2F-1* at transcriptional level in a dosage-dependent manner (Figure 6B). Taken together, we proposed that EcR-mediated ecdysone signaling is transcriptionally involved in the promotion of *E2F-1* and suppression of *CycE* in the cultured silkworm BmN4 cells.

### 2.5. Cell Cycle Genes Respond to Ecdysone Treatment in Silkworm Silk Gland

**Figure 7:**
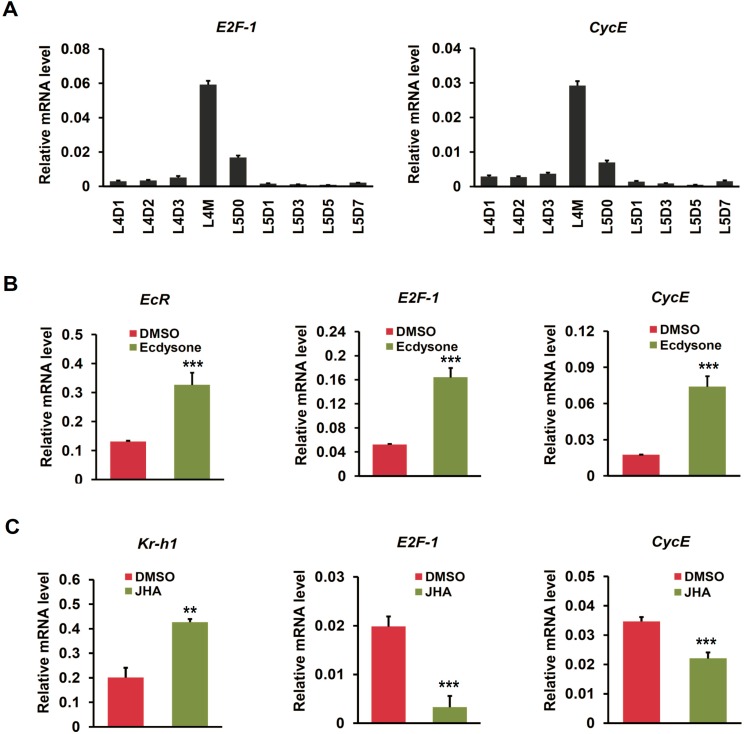
Ecdysone-induced changes in the expression of cell cycle genes in silkworm silk gland. (**A**) Expression patterns of cell cycle genes (*E2F-1* and *CycE*) in silk gland during silkworm larval molting. Nine developmental time points, including L4D1, L4D2, L4D3, L4M, L5D0, L5D1, L5D3, L5D5, and L5D7, were selected; (**B**) The expression changes of cell cycle genes (*E2F-1* and *CycE*) at 12 h after ecdysone treatment on 1-day-old fourth instar silkworm larvae were examined using quantitative RT-PCR. Error bars represents mean and S.D., ** *p* < 0.01, *** *p* < 0.001, compared with the control; (**C**) The expression changes of cell cycle genes (*E2F-1* and *CycE*) at 12 h after JHA treatment on 1-day-old fourth instar silkworm larvae were examined.

Our results revealed that ecdysone-mediated change of *CycE* expression in silkworm BmN4 cells was opposite to that of *E2F-1*. This is inconsistent with the observation in the fruit fly wing that *CycE* expression is positively regulated by E2F-1 [20,21]. To test whether ecdysone signaling in regulating the transcription of *E2F-1* and *CycE* may be different at cellular and individual scales, we examined the expression changes of these two cell cycle genes in silk gland of silkworm larvae with ecdysone treatment. Firstly, we noted that *E2F-1* and *CycE* were highly expressed during larval molting (Figure 7A), further indicating these two cell cycle genes were also transcriptionally regulated by ecdysone in silk gland. This is because ecdysone pulse is highly present during larval molting [38]. Furthermore, after ecdysone treatment, *EcR* was up-regulated, and both *E2F-1* and *CycE* were synchronously induced to be up-regulated (Figure 7B). Obviously, ecdysone-mediated up-regulation of *CycE* transcription in silkworm silk gland is consistent with the findings in the fruit fly wing, but opposite with that in the cultured BmN4 cells.

We also examined the transcriptional response of cell cycle genes in silk gland to JHA treatment. As shown in Figure 7C, JH responsive gene *Kr-h1* was up-regulated in silk gland of silkworm larvae with JHA treatment. Remarkably, in response to JHA, the transcriptions of both *E2F-1* and *CycE* were suppressed, which is completely converse with the effects of ecdysone. Taken together, we suggested that both the transcriptions of both *E2F-1* and *CycE* were positively regulated by ecdysone at individual scale.

### 2.6. Ecdysone Treatment Induces Cell Cycle Arrest in BmN4 Cells

**Figure 8:**
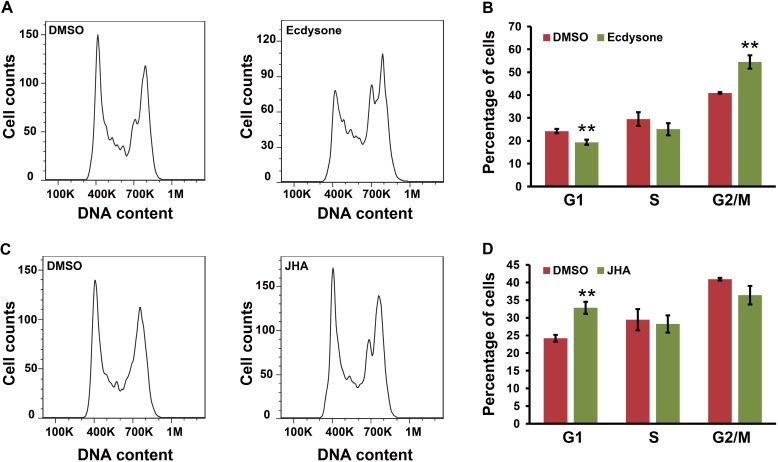
Ecdysone and JHA treatment induce cell cycle arrest. (**A**,**B**) Effect of ecdysone on cell cycle progression in BmN4 cells. At 24 h after ecdysone treatment, BmN4 cells were stained with Cell Cycle and Apoptosis Analysis Kit and subsequently detected by flow cytometry; (**C**,**D**) Effect of JHA on cell cycle progression in BmN4 cells. Similarly, at 24 h after JHA treatment, BmN4 cells were stained with Cell Cycle and Apoptosis Analysis Kit and subsequently detected by flow cytometry. Error bars represents mean and S.D., ** *p* < 0.01, compared with the control.

Undoubtedly, our findings raised a question why ecdysone regulation on *CycE* transcription was not positively correlated with its induction on *E2F-1* transcription in BmN4 cells. Previous reports have demonstrated that during normal cell cycle progression, *CycE* expression is periodically elevated during G1/S transition but is declined during G2/M transition [39,40], and *E2F-1* is expressed throughout the cell cycle and is dramatically increased in late G1 [41,42]. Therefore, we wonder whether the disruption of ecdysone signaling could induce cell cycle arrest, which in turn perturb the transcription of *CycE* transcription in BmN4 cells. To test this hypothesis, we further examined the effect of ecdysone treatment on cell cycle progression in BmN4 cells via flow cytometry analysis. As shown in Figure 8A,B, ecdysone treatment induced cell cycle arrest at G2/M phase, a time when *CycE* expression should be reduced as expected. The proportion of cells at G2/M phase was significantly increased from 40.9% in the control (DMSO treatment) to 54.5% after ecdysone treatment.

Furthermore, we analyzed the effect of JHA treatment on cell cycle progression in BmN4 cells. As shown in Figure 8C,D, JHA treatment induced cell cycle arrest at G1 phase, a time when *CycE* expression should be increased. A significant accumulation of cells at G1 phase could be observed. Taken together with the cell cycle-dependent changes of *CycE* expression, we speculated that although *E2F-1* transcription could be increased and decreased by ecdysone and JHA, respectively, the changes of *CycE* transcription after the treatment with either ecdysone or JHA is most possibly caused by cell cycle arrest induced by ecdysone or JHA, rather than E2F-1-dependent regulation.

## 3. Discussion

An increasing number of studies have reported that the ecdysone signaling pathway regulates cell cycle progression in insect. For example, the analysis in insect cell lines demonstrated that ecdysone application results in an accumulation of cells in the G1 phase in mosquito cells [43], and G2 arrest in the indianmeal moth (*Plodia interpunctella*) imaginal disc cells and fall armyworm (*Spodoptera frugiperda*) Sf9 cells [44,45,46]. *In vivo* analysis revealed that the ecdysone signaling pathway is required for cell cycle progression in the wing and eye disc of the fruit fly larvae [23,27,28,47]. Intriguingly, we found that EcR as ecdysone receptor is expressed in silkworm ovary-derived BmN4 cells at mRNA level. Although it is unclear how *EcR* transcription is activated in BmN4 cells, our investigation revealed that EcR-mediated ecdysone signaling is involved in cell cycle progression.

E2F-1 plays a crucial role in the regulation of cell cycle progression [48,49]. Previous studies in the fruit fly indicated that E2F-1 is likely a candidate target of EcR and is positively regulated by ecdysone at transcriptional level [23,50]. Intriguingly, based on cellular and individual analysis in silkworm, we found that *E2F-1* expression was up-regulated by *EcR* overexpression and ecdysone treatment in both cultured BmN4 cells and silk gland of larvae, but reduced by *EcR* RNAi and the treatment of JHA as an antagonist of ecdysone. This strongly supported that *E2F-1* transcription is positively regulated by EcR-mediated ecdysone signaling pathway.

As well known, CycE functions in controlling the G1/S transition during cell cycle progression [17], and *CycE* expression is positively regulated by E2F-1 in the fruit fly wing and salivary gland [20,21,51]. Our results showed that *CycE* expression was promoted after ecdysone treatment but suppressed after JHA application in silkworm silk gland. This is consistent with the changing tendency of *E2F-1* expression under the same condition, indicating that E2F-1 similarly contributes to regulate positively the transcription of *CycE* gene in silkworm silk gland. Curiously, in silkworm BmN4 cells, *CycE* expression was suppressed by ecdysone treatment but promoted by either JHA application or *EcR* RNAi, which was negatively associated with ecdysone or JHA-induced changes of *E2F-1* transcription.

It has been previously shown that *CycE* is highly expressed during G1/S transition and decreased during G2/M transition [39,40], whereas *E2F-1* transcription continues throughout the cell cycle [41,42]. Our further examination demonstrated that ecdysone and JHA could induce cell cycle arrest at G2/M and G1 phases in BmN4 cells, respectively. This appears to be well synchronized with decreased transcription of *CycE* after ecdysone treatment and increased transcription of *CycE* after JHA treatment. Taken together with the fact of cell cycle-dependent changes of *CycE* transcription, we proposed that unlike *E2F-1*, ecdysone-mediated changes of *CycE* transcription in BmN4 cells is due most possibly to the response to ecdysone-induced cell cycle arrest, rather than E2F-1 regulation from ecdysone signaling cascades. Undoubtedly, the detailed mechanism underlying the regulation of EcR-mediated ecdysone signaling on *CycE* transcription needs to be further elucidated.

## 4. Experimental Section

### 4.1. Cell Lines

The silkworm cell lines of BmN4 and BmN4-SID1 were used in our experiment. The BmN4 cell line was derived from the silkworm ovary and the BmN4-SID1 cell line was established by introducing the nematode (*Caenorhabditis elegans*) SID1 gene into BmN4 cells, which can greatly enhance the uptake of exogenous dsRNA [33]. The BmN4-SID1 and BmN4 cell line were used to check the effect of *EcR* RNAi and hormone treatment on the transcription of cell cycle genes, respectively. The BmN4 and BmN4-SID1 cell lines were cultured at 27 °C in IPL-41 medium.

### 4.2. RNAi Knockdown of EcR Gene in BmN4-SID1 Cells

RNAi knockdown of *EcR* gene in BmN4-SID1 cells was performed according to the previously described approach [31]. In summary, the dsRNAs against silkworm *EcR* gene and EGFP (enhanced green fluorescent protein; dsEGFP) were separately synthesized and transfected into BmN4-SID1 cells. We collected the BmN4-SID1 cells at the 7th day after dsRNA treatment for twice to check cell shape using microscope and examined expression change of the selected genes.

### 4.3. RT-PCR Assay

Semi-quantitative and quantitative RT-PCR experiments were performed to determine whether the selected genes were expressed in BmN4 cells. RNA extraction, cDNA synthesis, and RT-PCR reaction were carried out following previous reports [52]. The silkworm ribosomal protein L3 (*RpL3*) gene was selected as a control. The primers used here are listed in Table S1.

### 4.4. Overexpression of EcR Gene in BmN4 Cells

For the overexpression of silkworm *EcR* in BmN4 cells, the opening reading frame sequence of silkworm *EcR* gene was cloned into the expression vector pSL1180. The constructs was transfected into BmN4 cells in 12-well plates. At 72 h after transfection, the BmN4 cells were collected to extract RNA for RT-PCR-based expression pattern analysis.

### 4.5. Hormone Treatment in BmN4 Cells

The BmN4 cells were seeded into 12-well plates with approximately 10^6^ cells per well and cultured for 12 h. After a replacement with 1 mL fresh medium, the cells were subsequently treated with different dosage of either ecdysone or JHA. The treated BmN4 cells were cultured for 24 h at 27 °C and were collected for further RT-PCR analysis. In addition, for a time-dependent analysis, the BmN4 cells were treated with 1 μg per well and were collected at 6, 12, 24 and 48 h after hormone treatment.

### 4.6. Gene Expression Assay in Silkworm Silk Gland

The silkworm strain *Dazao* were reared with fresh mulberry leaves under the conditions of 25 °C and 12 h light/12 h dark photoperiod. The silk gland was isolated at nine developmental time points, including L4D1 (Day 1 of the fourth larval instar), L4D2, L4D3, L4M (fourth larval molting), L5D0 (0 h after fourth molts), L5D1 (day 1 of the fifth larval instar), L5D3, L5D5, and L5D7, and used for further RT-PCR analysis. Moreover, ecdysone and JHA with the dosage of 1 μg per individual were separately injected into silkworm larvae on Day 1 of the fourth instar. DMSO treatment was used as a control. At 12 h after hormone treatment, silk gland was isolated for further RT-PCR analysis.

### 4.7. Cell Cycle Analysis by Flow Cytometry

The BmN4 cells that were seeded into 12-well plate with 10^6^ cells per well in 1 mL medium were used for cell cycle analysis. After culturing for 12 h, the cells were treated by ecdysone or JHA with a dosage of 1 μg per well, respectively. DMSO treatment was used as a control. At 24 h after treatment, the cells were stained with Cell Cycle and Apoptosis Analysis Kit (Beyotime Biotechnology, Nantong, China) and subsequently analyzed on flow cytometer (Becton, Dickinson and Company, Franklin Lakes, NJ, USA). Data was analyzed using Flow Jo 7.6 software (Flowjo, Ashland, OR, USA).

## 5. Conclusions

In this study, we demonstrated at cellular and individual scales that the EcR-mediated ecdysone signaling pathway is involved in regulating the transcription of two cell cycle genes, *E2F-1* and *CycE*, in the silkworm. It is expected to facilitate the future work about deciphering the roles of EcR-mediated ecdysone signaling in cell cycle progression in insect.

## Supplementary Materials

Supplementary materials can be found at http://www.mdpi.com/1422-0067/16/02/3335/s1.

## References

[1] Koelle M.R., Talbot W.S., Segraves W.A., Bender M.T., Cherbas P., Hogness D.S. (1991). The *Drosophila* EcR gene encodes an ecdysone receptor, a new member of the steroid receptor superfamily. Cell.

[2] Fahrbach S.E., Smagghe G., Velarde R.A. (2012). Insect nuclear receptors. Annu. Rev. Entomol..

[3] King-Jones K., Thummel C.S. (2005). Nuclear receptors—A perspective from *Drosophila*. Nat. Rev. Genet..

[4] Andres A.J., Thummel C.S. (1992). Hormones, puffs and flies: The molecular control of metamorphosis by ecdysone. Trends Genet..

[5] Thummel C.S. (1996). Flies on steroids—*Drosophila* metamorphosis and the mechanisms of steroid hormone action. Trends Genet..

[6] Yamanaka N., Rewitz K.F., O’Connor M.B. (2013). Ecdysone control of developmental transitions: Lessons from *Drosophila* research. Annu. Rev. Entomol..

[7] Carney G.E., Wade A.A., Sapra R., Goldstein E.S., Bender M. (1997). DHR3, an ecdysone-inducible early-late gene encoding a *Drosophila* nuclear receptor, is required for embryogenesis. Proc. Natl. Acad. Sci. USA.

[8] Li T., Bender M. (2000). A conditional rescue system reveals essential functions for the ecdysone receptor (EcR) gene during molting and metamorphosis in *Drosophila*. Development.

[9] Baehrecke E.H. (1996). Ecdysone signaling cascade and regulation of *Drosophila* metamorphosis. Arch. Insect Biochem. Physiol..

[10] Schwedes C.C., Carney G.E. (2012). Ecdysone signaling in adult *Drosophila melanogaster*. J. Insect Physiol..

[11] Tapon N., Moberg K.H., Hariharan I.K. (2001). The coupling of cell growth to the cell cycle. Curr. Opin. Cell Biol..

[12] Levine E. (2004). Cell cycling through development. Development.

[13] Neufeld T.P., Edgar B.A. (1998). Connections between growth and the cell cycle. Curr. Opin. Cell Biol..

[14] Swanhart L., Kupsco J., Duronio R.J. (2005). Developmental control of growth and cell cycle progression in *Drosophila*. Methods Mol. Biol..

[15] Sanchez I., Dynlacht B.D. (2005). New insights into cyclins, CDKs, and cell cycle control. Semin. Cell Dev. Biol..

[16] Lim S., Kaldis P. (2013). Cdks, cyclins and CKIs: Roles beyond cell cycle regulation. Development.

[17] Knoblich J.A., Sauer K., Jones L., Richardson H., Saint R., Lehner C.F. (1994). Cyclin E controls S phase progression and its down-regulation during *Drosophila* embryogenesis is required for the arrest of cell proliferation. Cell.

[18] Edgar B.A., Lehman D.A., O’Farrell P.H. (1994). Transcriptional regulation of string (cdc25): A link between developmental programming and the cell cycle. Development.

[19] Nigg E.A. (1995). Cyclin-dependent protein kinases: Key regulators of the eukaryotic cell cycle. BioEssays.

[20] Neufeld T.P., de la Cruz A.F., Johnston L.A., Edgar B.A. (1998). Coordination of growth and cell division in the *Drosophila* wing. Cell.

[21] Reis T., Edgar B.A. (2004). Negative regulation of dE2F1 by cyclin-dependent kinases controls cell cycle timing. Cell.

[22] Edgar B.A., Lehner C.F. (1996). Developmental control of cell cycle regulators: A fly’s perspective. Science.

[23] Cranna N., Quinn L. (2009). Impact of steroid hormone signals on *Drosophila* cell cycle during development. Cell Div..

[24] Ninov N., Manjon C., Martin-Blanco E. (2009). Dynamic control of cell cycle and growth coupling by ecdysone, EGFR, and PI3K signaling in *Drosophila* histoblasts. PLoS Biol..

[25] Stevens B., Alvarez C.M., Bohman R., O’Connor J.D. (1980). An ecdysteroid-induced alteration in the cell cycle of cultured *Drosophila* cells. Cell.

[26] Fallon A.M., Gerenday A. (2010). Ecdysone and the cell cycle: Investigations in a mosquito cell line. J. Insect Physiol..

[27] Mitchell N.C., Lin J.I., Zaytseva O., Cranna N., Lee A., Quinn L.M. (2013). The Ecdysone receptor constrains wingless expression to pattern cell cycle across the *Drosophila* wing margin in a Cyclin B-dependent manner. BMC Dev. Biol..

[28] Mitchell N., Cranna N., Richardson H., Quinn L. (2008). The Ecdysone-inducible zinc-finger transcription factor Crol regulates Wg transcription and cell cycle progression in *Drosophila*. Development.

[29] Johnston L.A., Edgar B.A. (1998). Wingless and Notch regulate cell-cycle arrest in the developing *Drosophila* wing. Nature.

[30] Johnston L.A., Sanders A.L. (2003). Wingless promotes cell survival but constrains growth during *Drosophila* wing development. Nat. Cell Biol..

[31] Li Z., Cheng D., Mon H., Tatsuke T., Zhu L., Xu J., Lee J.M., Xia Q., Kusakabe T. (2012). Genome-wide identification of polycomb target genes reveals a functional association of Pho with Scm in *Bombyx mori*. PLoS One.

[32] Jayachandran G., Fallon A.M. (2000). Evidence for expression of EcR and USP components of the 20-hydroxyecdysone receptor by a mosquito cell line. Arch. Insect Biochem. Physiol..

[33] Mon H., Kobayashi I., Ohkubo S., Tomita S., Lee J., Sezutsu H., Tamura T., Kusakabe T. (2012). Effective RNA interference in cultured silkworm cells mediated by overexpression of *Caenorhabditis elegans* SID-1. RNA Biol..

[34] Truman J.W., Riddiford L.M. (2002). Endocrine insights into the evolution of metamorphosis in insects. Annu. Rev. Entomol..

[35] Dubrovsky E.B. (2005). Hormonal cross talk in insect development. Trends Endocrinol. Metab..

[36] Minakuchi C., Zhou X., Riddiford L.M. (2008). Kruppel homolog 1 (Kr-h1) mediates juvenile hormone action during metamorphosis of *Drosophila melanogaster*. Mech. Dev..

[37] Kayukawa T., Minakuchi C., Namiki T., Togawa T., Yoshiyama M., Kamimura M., Mita K., Imanishi S., Kiuchi M., Ishikawa Y. (2012). Transcriptional regulation of juvenile hormone-mediated induction of Kruppel homolog 1, a repressor of insect metamorphosis. Proc. Natl. Acad. Sci. USA.

[38] Mizoguchi A., Ohashi Y., Hosoda K., Ishibashi J., Kataoka H. (2001). Developmental profile of the changes in the prothoracicotropic hormone titer in hemolymph of the silkworm *Bombyx mori*: Correlation with ecdysteroid secretion. Insect Biochem. Mol. Biol..

[39] Siu K.T., Rosner M.R., Minella A.C. (2012). An integrated view of cyclin E function and regulation. Cell Cycle.

[40] Keyomarsi K., Herliczek T.W. (1997). The role of cyclin E in cell proliferation, development and cancer. Prog. Cell Cycle Res..

[41] Johnson D.G., Ohtani K., Nevins J.R. (1994). Autoregulatory control of E2F1 expression in response to positive and negative regulators of cell cycle progression. Genes Dev..

[42] Adams M.R., Sears R., Nuckolls F., Leone G., Nevins J.R. (2000). Complex transcriptional regulatory mechanisms control expression of the E2F3 locus. Mol. Cell. Biol..

[43] Gerenday A., Fallon A.M. (2004). Ecdysone-induced accumulation of mosquito cells in the G1 phase of the cell cycle. J. Insect Physiol..

[44] Siaussat D., Bozzolan F., Porcheron P., Debernard S. (2008). The 20-hydroxyecdysone-induced signalling pathway in G2/M arrest of Plodia interpunctella imaginal wing cells. Insect Biochem. Mol. Biol..

[45] Giraudo M., Califano J., Hilliou F., Tran T., Taquet N., Feyereisen R., le Goff G. (2011). Effects of hormone agonists on Sf9 cells, proliferation and cell cycle arrest. PLoS One.

[46] Siaussat D., Bozzolan F., Queguiner I., Porcheron P., Debernard S. (2005). Cell cycle profiles of EcR, USP, HR3 and B cyclin mRNAs associated to 20E-induced G2 arrest of *Plodia interpunctella* imaginal wing cells. Insect Mol. Biol..

[47] Zelhof A.C., Ghbeish N., Tsai C.C., Evans R.M., McKeown M. (1997). A role for ultraspiracle, the *Drosophila* RXR, in morphogenetic furrow movement and photoreceptor cluster formation. Development.

[48] Sala A., Nicolaides N.C., Engelhard A., Bellon T., Lawe D.C., Arnold A., Grana X., Giordano A., Calabretta B. (1994). Correlation between E2f-1 requirement in the S-Phase and E2f-1 transactivation of cell cycle-related genes in human cells. Cancer Res..

[49] Duronio R.J., Ofarrell P.H., Xie J.E., Brook A., Dyson N. (1995). The transcription factor E2f is required for S-Phase during *Drosophila* embryogenesis. Gene Dev..

[50] Gauhar Z., Sun L.V., Hua S., Mason C.E., Fuchs F., Li T.R., Boutros M., White K.P. (2009). Genomic mapping of binding regions for the Ecdysone receptor protein complex. Genome Res..

[51] Duronio R.J., Ofarrell P.H. (1995). Developmental control of the G(1) to S-transition in *Drosophila*—Cyclin-E Is a limiting downstream target of E2f. Gene Dev..

[52] Li Z., Tatsuke T., Sakashita K., Zhu L., Xu J., Mon H., Lee J.M., Kusakabe T. (2012). Identification and characterization of Polycomb group genes in the silkworm, *Bombyx mori*. Mol. Biol. Rep..

